# Reusing excess staple oligonucleotides for economical production of DNA origami

**DOI:** 10.1093/nar/gkaf527

**Published:** 2025-06-18

**Authors:** Giorgia Isinelli, Christopher M Wintersinger, Matthew Aquilina, Pascal Lill, Olivia J Young, Jie Deng, William M Shih, Yang C Zeng

**Affiliations:** Department of Cancer Biology, Dana–Farber Cancer Institute, Harvard Medical School, Boston, MA 02115, United States; Wyss Institute for Biologically Inspired Engineering at Harvard University, Boston, MA 02115, United States; Department of Biological Chemistry and Molecular Pharmacology, Harvard Medical School, Boston, MA 02115, United States; Department of Drug Science and Pharmacology, University of Catania, Catania 95125, Italy; Department of Cancer Biology, Dana–Farber Cancer Institute, Harvard Medical School, Boston, MA 02115, United States; Wyss Institute for Biologically Inspired Engineering at Harvard University, Boston, MA 02115, United States; Department of Biological Chemistry and Molecular Pharmacology, Harvard Medical School, Boston, MA 02115, United States; Department of Cancer Biology, Dana–Farber Cancer Institute, Harvard Medical School, Boston, MA 02115, United States; Wyss Institute for Biologically Inspired Engineering at Harvard University, Boston, MA 02115, United States; Department of Biological Chemistry and Molecular Pharmacology, Harvard Medical School, Boston, MA 02115, United States; Wyss Institute for Biologically Inspired Engineering at Harvard University, Boston, MA 02115, United States; Wyss Institute for Biologically Inspired Engineering at Harvard University, Boston, MA 02115, United States; Department of Cancer Biology, Dana–Farber Cancer Institute, Harvard Medical School, Boston, MA 02115, United States; Department of Biological Chemistry and Molecular Pharmacology, Harvard Medical School, Boston, MA 02115, United States; Department of Cancer Biology, Dana–Farber Cancer Institute, Harvard Medical School, Boston, MA 02115, United States; Wyss Institute for Biologically Inspired Engineering at Harvard University, Boston, MA 02115, United States; Department of Biological Chemistry and Molecular Pharmacology, Harvard Medical School, Boston, MA 02115, United States; Department of Cancer Biology, Dana–Farber Cancer Institute, Harvard Medical School, Boston, MA 02115, United States; Wyss Institute for Biologically Inspired Engineering at Harvard University, Boston, MA 02115, United States; Department of Biological Chemistry and Molecular Pharmacology, Harvard Medical School, Boston, MA 02115, United States

## Abstract

DNA origami has enabled the development of responsive drug-delivery vehicles with precision features that were previously not attainable in bionanotechnology. To reduce the costs of creating therapeutic-scale amounts of DNA origami that need to bear costly modifications with high occupancy, we reused the excess staple oligonucleotides that are left over from the folding process to fold additional origami. We determined that a DNA origami can be successfully folded with up to 80% cost savings by cyclic recovery and reuse of excess staple strands. We found evidence that higher-quality staple strands are preferentially incorporated into origami, consistent with past reports, and therefore are preferentially depleted from the free-strand pool. The folding of DNA origami with staple strands that were reused up to 11 times was indistinguishable by our panel of assays versus a control folded with new strands, so long as the reused oligonucleotides were replenished each cycle with a small excess of fresh strands. We also continued to observe a high degree of cargo loading [e.g. oligo with PS backbone modification (CpG), fluorophores (Cy5 or Cy3), smaller nanostructures (nanocube), etc.] on the origami with each folding cycle. By recovering, reusing, and replenishing excess staple oligonucleotides, it is possible to significantly lessen production costs to create well-formed origami, which is useful to allow more therapeutic designs to be tested.

## Graphical Abstract

**Graphical Abstract:**
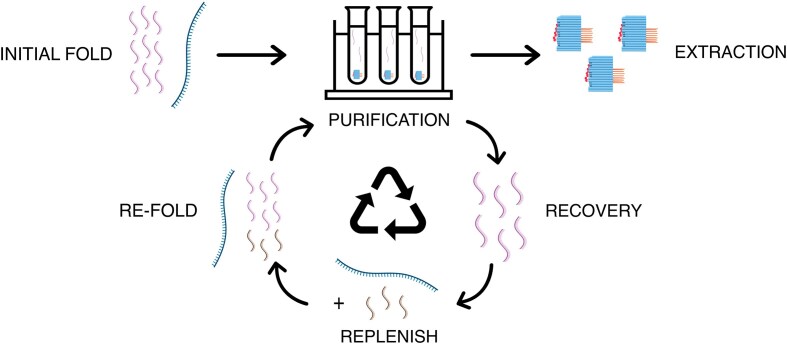

## Introduction

DNA origami is a proven and robust method for forward design and synthesis of two- and three-dimensional nanoscale shapes for responsive drug-delivery vehicles [1]. In the approach, an excess of complementary “staple” DNA oligonucleotides [2] anneal to a long single-stranded DNA (ssDNA) “scaffold,” with strategically placed crossovers to fold the scaffold into the designed shape. One promising application of origami is as vaccines [3–5] and for drug delivery, where the terminal ends of staple oligonucleotides are used to precisely position therapeutic cargoes at nanometer spacing intervals [3, 6, 7]. Such programmability has enabled drug-delivery vehicles that release their payload when triggered by *in vivo* stimuli and vaccines with tunable immune responses [8–10]. However, the therapeutic use of origami has been impeded by a number of challenges, including two key ones that need to be considered together, highlighted below.

First, the cost of manufacture of large amounts of origami bearing staple strands with expensive modifications presents an impediment for its application as therapeutics [11]. This expense is amplified by the requirement for providing larger excesses of staple strands over scaffold to ensure the highest incorporation efficiency. Milligram scales of product as needed to prototype potential therapeutics are most readily made using staple oligonucleotides from chemical-synthesis vendors, which typically represent the majority of the material cost of DNA-origami fabrication (see Supplementary Text S1.1 for further discussion from Supplementary Table S2 to Supplementary Table S5).

Second, reliable and repeatable results with therapeutic origami require folding conditions that favor high incorporation rates of the designed features. It is notable that the robustness of origami folding, in terms of preferential incorporation of higher-quality staple strands, allows production of well-formed origami even with unpurified commercially purchased staple strands. For instance, a typical commercial synthesis with solid support phosphoramidite chemistry yields upward of ∼0.99^( *n*−1)^ for an *n* length strand. For a typical 42-mer staple, about one-third of the product strands are not synthesized to their full length. Thus, one-third of staples designed to include an attached 5′ cargo molecule would be missing their critical payload. Despite this, folding is resilient to impurities of the staple-strand pool because of cooperative processes favoring the addition of full-length strands. Nonetheless, there is still only ∼50%–95% incorporation rate of any particular 3′ feature using typical reaction conditions, in the case of folding with a 10-fold excess of staple strands [12]. Larger staple excesses or purification of the input oligonucleotides can further improve the incorporation of desired 3′ and 5′ features, but this would require greater amounts of starting materials and therefore would further increase the cost of production for applications such as therapeutics.

**Figure 1.**
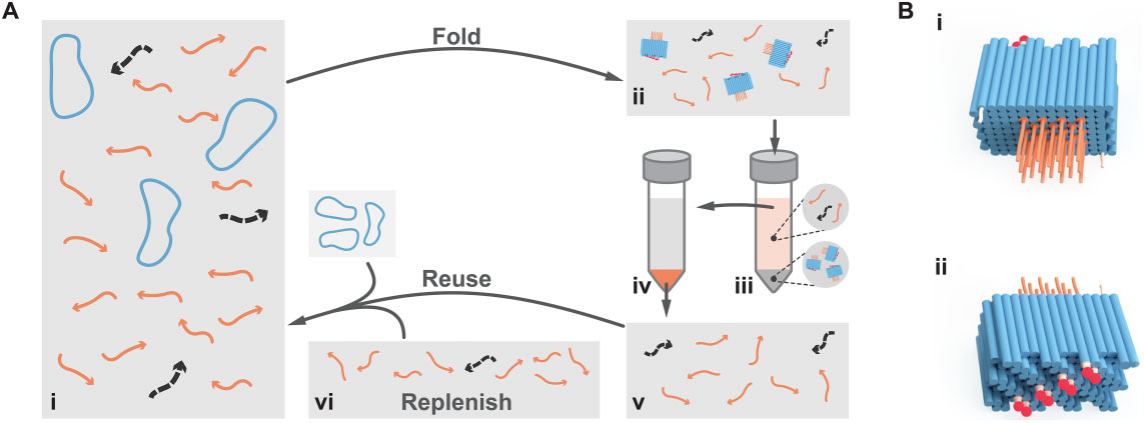
Proof-of-concept therapeutic DNA origami square block (SQB) and an approach for folding it by reusing excess staple strands. (**A**) (i) Folding mixture with the circular scaffold DNA 50 nM (blue) and excess unpurified staple strands (0.25 μM core staples, 0.5 μM handles, and 1 μM for functional staples), where full-length strands are solid arrows (orange) versus truncated impurity strands are dotted arrows (dark gray). (ii) After folding, the solution contains SQB origami and excess staples. (iii) PEG precipitation is used to separate the DNA origami (in the pellet) from the unused staple strands (in the supernatant). (iv) Ethanol precipitation followed by centrifugation is used to extract the unused staple strands (into the pellet) from the PEG purification buffer and remove any residual polyethylene glycol (PEG) from solution. (v) The folding process favors incorporation of full-length strands into the origami over their truncated counterparts, reducing the average purity of the remaining strand pool. (vi) The recovered strands may be reused to fold more DNA origami, if they are replenished with an equivalent of strands to what were consumed in the preceding folding experiment. We posit that replenishing the reused strands maintains full-length strands at sufficient concentration, such that staple strands may be reused multiple cycles to fold high-quality origami. (**B**) (i, ii) Renderings of the front and back cargo-holding faces of the DNA origami square block with CpG and fluorophores conjugated.

To address this unmet need for lower-cost origami therapeutics, here we have explored reusing the excess staple strands left over from folding to synthesize more origami in the context of milligram-scale production suitable for animal studies (Fig. 1A). This can yield substantial savings on material cost for staple strands, with greater cost reductions as excess strands are reused repeatedly to fold additional rounds of origami. With a 10-fold excess of staple strands in the folding reaction, the unit cost of oligonucleotides for the origami could be nearly halved with only a single cycle of staple reuse, versus using only freshly purchased staple strands. Moreover, the total oligonucleotide costs could be lessened by ∼80% with 10 rounds of reuse or when larger excesses of staple strands are used (Supplementary Fig. S1 and Supplementary Text S1.2). Given that folding with excess staples is a necessary step for almost every DNA origami design, the impact of staple reuse could be immediately realized with both small- and large-scale methodologies [13]. In particular, workflows utilizing expensive chemically modified staples are excellent targets for substantial cost savings (Supplementary Text S1).

We initially observed that the staple strands left over from folding could be efficiently recovered by ethanol precipitation from a supernatant containing the remaining staple strands after recovery of the origami product by PEG precipitation (Supplementary Fig. S2; see the “PEG precipitation to purify origami from excess staples” and “Recovery of excess staple oligonucleotides from PEG supernatant” sections). Encouraged by this technique, we tested whether the reuse of staple strands would allow the formation of DNA origami designed for a therapeutic application with satisfactory incorporation of 3′ and 5′ features on the product.

One pitfall we foresaw was that the relative purity of the staple strands might be progressively lessened with reuse if folding preferentially incorporates full-length staple strands [2], or if long incubations at elevated temperatures lead to accumulation of significant depurination event [14]. We hypothesized that replenishment of the recovered staple pool with fresh staple strands, to replace each equivalent of staple strands that were removed in the prior folding cycle, would sufficiently restore the average strand quality to an extent that enables folding of additional origami of similar quality to those from earlier cycles (Fig. 1A, vi, and Supplementary Fig. S3; see Supplementary Text S1.1 for discussion about the DNA origami costs).

## Materials and methods

### SQB DNA origami folding

Unpurified dehydrated staple oligonucleotides were purchased from Integrated DNA Technologies (IDT) at either 10- or 100-nmol scales and rehydrated in water so that the concentration of each strand was ∼0.2 or ∼0.5 mM. Oligos for a particular origami were combined using volumes to create 2× staple strand stocks, with the concentrations of each strand specified in Supplementary Table S1. The p8634 scaffold strand was produced from M13 phage replication in JM109 *Escherichia coli*. Large-scale (∼40 ml) folding mixtures were prepared in 10 parts total as follows: 5 parts of the 2× staple strand stock, 1 part folding buffer [50 mM Tris, pH 8.0, 10 mM ethylenediaminetetraacetic acid (EDTA), 120 mM MgCl_2_], with the remaining 4 parts to add the appropriate scaffold at 50 nM final concentration, Cy5 labeling strand at the concentration noted in Supplementary Table S1, and water. The larger reaction mixture was split into smaller volumes in 0.2-mL PCR tubes and incubated on a thermocycler as follows: 80°C for 15 min in a single step; 50–40°C in 100 10.8 min steps reducing by 0.1°C/step; 4°C thereafter until collection of the sample for further processing and PEG purification [6].

### Barrel DNA origami folding

Unpurified dehydrated staple oligonucleotides were purchased from IDT at either 10- or 100-nmol scales and rehydrated in water so that the concentration of each strand was ∼0.2 or ∼0.5 mM. Oligos for a particular origami were combined using volumes to create 2× staple strand stocks, with the concentrations of each strand specified in Supplementary Table S6. The p8634 scaffold strand was produced from M13 phage replication in JM109 *E. coli*. DNA origami folding mixtures were prepared in 10 parts total as follows: 5 parts of the 2× staple stock, 1 part of folding buffer (50 mM Tris, pH 8.0, 10 mM EDTA, 120 mM MgCl_2_), with the remaining 4 parts to add the appropriate scaffold at 50 nM final concentration, Cy5 labeling strand at the concentration noted in Supplementary Table S6, and water. The larger reaction mixture was split into smaller volumes in 0.2-ml PCR tubes to be incubated on a thermocycler as follows: 80°C for 15 min; decrease the temperature to 60–25°C in 350 3.08 min steps reducing by 0.1°C/step; 4°C thereafter until collection of the sample for further processing and PEG purification [15].

### Nanocube folding and purification

The design for a 10 nm × 10 nm × 10 nm nanocube was adapted from a previously published version [16]. Unpurified dehydrated nanocube oligonucleotides were purchased from IDT at a 10-nmol scale, rehydrated in water at ∼100 μM each, and mixed with equal volumes per strand. Out of the 28 total nanocube strands, one strand was selected and appended with a 4T linker and 16-nt handle to its 3′ end. The nanocube was prepared with ∼1 μM of each strand (except for the handle tagged strand at ∼2 μM), 40 mM MgCl_2_, and folded with a 42-h temperature gradient: 80°C for 10 min in a single step; 65–37°C in 290 8.69 min steps reducing by 0.1°C/step; 16°C thereafter until collection of the sample. The purification method used was not the one indicated in the original article because it provided a solution with a yield that was too low for the experiments. The nanocubes have been precipitated with 20% PEG precipitation in a 1:1 ratio volume, MgCl_2_ balanced. The resulting pellet was resuspended keeping into consideration that for the experiment the concentration had to be high enough. Then, we performed agarose-gel-based extraction. With this combined method, a concentrated solution of pure product was obtained.

### PEG precipitation to purify origami from excess staple strands

The following protocol was adapted from as previously published [17]. For the SQB, one volume of a PEG purification buffer [5 mM Tris, pH 8.0, 1 mM EDTA, 15% (w/v) PEG-8000, 510 mM NaCl, 8 mM MgCl_2_] was added to an equal volume of the raw folded SQB in a 50-ml conical tube. For the barrel, the raw folding reaction was diluted 10-fold (in 5 mM Tris, 1 mM EDTA, 12 mM MgCl_2_). Next, one volume of diluted barrel folding reaction was added to 10 volumes of a PEG purification buffer [5 mM Tris, 1 mM EDTA, 10% (w/v) PEG-8000, 510 mM NaCl, ∼12 mM MgCl_2_] in a 15-ml conical tube. The sample was mixed thoroughly by aspirating and dispensing it using a pipette, incubated for 30 min in a dark setting, and then placed on a centrifuge at 16 000 × *g* for 25 min at room temperature. The majority of the supernatant was separated from the pellet containing the SQB by either decanting or removing it with a pipette. Next, the sample was centrifuged for an additional 2 min at 16 000 × *g* so that the remaining supernatant could be gently extracted using a pipette. We note that the pellets appeared blue, because of the Cy5 fluorophore attached to the SQB. A volume of buffer (5 mM Tris, pH 8.0, 1 mM EDTA, 10 or 12 mM MgCl_2_ for the SQB and barrel, respectively) was added to dilute the origami at the approximate desired concentration and incubate for 5 min at 37°C. The pellet was detached from the wall of the tube by flicking it, at which point the sample was placed on a shaker at 600 rpm, 37°C for 25 min. The sample was cooled to room temperature and then the origami concentration was determined by measuring the amount of DNA on a NanoDrop 2000c spectrophotometer.

### Recovery of excess staple oligonucleotides from PEG supernatant

The leftover staple supernatant from PEG purification was reserved from the last step of the “PEG precipitation to purify origami from excess staples” section. We recorded the total volume of supernatant and used a NanoDrop 2000c spectrophotometer to quantify the starting amount of ssDNA. In four parts total, one part of staple supernatant was mixed thoroughly with three parts of ethanol (100% or 200-proof). Next, 1/10th of the staple supernatant volume of 3 M sodium acetate was added. The solution was cooled in a −80°C freezer for 1 h and then put in a chilled centrifuge at 21 000 × *g* for 30 min. The majority of ethanol supernatant was decanted from the tube into another one. Then, the tube was briefly centrifuged so that the last traces of supernatant could be removed with a pipette. We proceeded to wash the pellet with a 1/10th of the total previous volume with cold ethanol (75%, v/v). Then, the tube was centrifuged for 5 min at the same speed and temperature as the previous step. The ethanol was decanted and removed with a pipette as described above, and the wash step was repeated one more time. The pellet was left to air dry in a dust-free place, with the total drying time selected depending on the relative size of the pellet (i.e. 15 min for small pellets versus overnight for the largest pellets). Sterile water was added to the dried pellet in a volume to dilute the staple strands to the approximate desired concentration. The final concentration of the strands was determined on a NanoDrop 2000c spectrophotometer, such that the recovery yield could be determined with respect to the starting measurement.

We note that this yield was necessary for the amounts of replenishing strands in the “Typical approach for replenishment of the recovered staples” section. Also, salt contamination of the recovered staple strands was problematic for the successful folding of the SQB. In cases where the 260/230 absorbance ratio was much larger than ∼2.2, we concluded that contaminating salts of the oligonucleotides were too high. In these occurrences, ethanol precipitation of the sample was repeated as above until a satisfactory 260/230 ratio was attained.

### Typical approach for replenishment of the recovered staple strands

This approach is appropriate if certain staple strands have different relative excesses versus other staple strands with respect to the scaffold, in a given folding mixture. Recovered staple strands from the “Recovery of excess staple oligonucleotides from PEG supernatant” section were replenished with an equivalent amount of fresh strands to replace strands extracted by the origami in the prior folding or that were lost in the recovery procedure. The following considerations were made in replenishing the staple strands: (i) each type of SQB staple is added in differing stoichiometric excesses with respect to the scaffold (Supplementary Table S1); (ii) the scaffold during the previous folding removed one equivalent of staple strands from the total strand excess; and (iii) there was a marginal loss of staple strands during recovery in the “Recovery of excess staple oligonucleotides from PEG supernatant” section. As such, the volume of each staple type mixture to add for replenishment was determined as below. The original_volume refers to the volume added initially to create the 2× staple strand stock during the initial folding with fresh strands in the “SQB DNA origami folding” section and the recovery_yield was determined in “Recovery of excess staple oligonucleotides from PEG supernatant” section.


\begin{eqnarray*}
{\rm volume}{\mathrm{\ }} = {\mathrm{\ }}{\rm original}\_{\rm volume}\_{\rm added}{\mathrm{\ *\ }}\left( {1 - {\mathrm{\ }}{\rm recovery}\_{\rm yield}{\mathrm{\ }} + {\mathrm{\ }}\frac{{{\rm recovery}{\rm }\_{\rm yield}}}{{{\rm excess}{\rm }}}} \right).
\end{eqnarray*}

The replenishing strands were added to the recovered staple mixture in the volumes as computed above, with the mixture diluted with water to attain the 2× staple strand stock volume that was added during reaction setup for the previous folding. We proceeded to set up the folding reaction using the replenished staple mixture as described in the “SQB DNA origami folding” section. Additionally, we added one-fifth of the amount of Cy5 labeling strand initially used in the “SQB DNA origami folding” section.

### Alternative approach for replenishment of the staple strands

This approach is appropriate if all the staple strands have the same relative excesses with respect to the scaffold in a given folding mixture. Initially, we used a NanoDrop 2000c spectrophotometer to determine the initial ssDNA concentration of the 2× staple stock mixture used in the “SQB DNA origami folding” section. The excess staple strands as recovered in “Recovery of excess staple oligonucleotides from PEG supernatant” section were rehydrated in a minimal volume of sterile water, and were diluted in more water to match the concentration of the initial 2× staple stock mixture. The final volume of the recovered diluted strands was recorded, with an additional fresh 2× staple stock mixture added to restore the volume of staple stock used during the prior folding cycle. Finally, we prepared the folding reaction with the replenished staple mixture as described in the “SQB DNA origami folding” section.

### Negative-stain transmission electron microscopy

Origami samples were diluted to a final concentration of 4 nM in 1× TEF buffer (5 mM Tris, pH 8.0, 1 mM EDTA, 10–12 mM MgCl_2_). For the SQB samples we used a 1× TEF buffer with 10 mM MgCl_2_ to dilute. For the nanocube samples alone and the SQBs conjugated with nanocubes, we used a 1× TEF buffer with 10 mM MgCl_2_. For the barrel samples, we used 1× TEF buffer with 12 mM MgCl_2_ to dilute and for the conjugated product barrel–SQB we used 1× TEF buffer with 11 mM MgCl_2_. TEM grids (Electron Microscopy Sciences FCF400-CU-50 or alternatively grids that we carbon-coated ourselves) were negatively glow discharged at 15 mA for 25 s in a PELCO easiGlow. The diluted sample (4 μl) was applied to the glow discharged grid, incubated for 2 min, and wicked off completely into Whatman paper (Fisher Scientific, 09-874-16B). Immediately after that, 10 μl of 2% aqueous filtered uranyl formate was applied for 1–2 s, and then immediately wicked off. The procedure was repeated and this time the uranyl formate was left for 40–45 s and then dried against Whatman paper leaving a very thin layer on the grid. Grids were left to dry in a dust-free area. All imaging was performed at 80 kV on a JEOL JEM 1400 plus microscope and captured with AMT Image Capture Engine Software Version 7.0.0.255. Micrographs were collected at a nominal magnification of 30k and pixel size 0.4 nm/px for SQB samples conjugated with barrels, and for SQBs conjugated with nanocubes the typical magnification was 40k with 0.3 nm/px as pixel size. Conjugation analysis of cargoes to the SQB was performed using the EMAN 2.11 software package [18]. Particles were selected and extracted using the e2boxer tool and visualized using e2display.

All images presented in this work were imported into FIJI ImageJ (v1.53c) (https://imagej.net/ij/index.html), corrected for background noise using a pseudo-flat-field image, and then contrast and brightness were adjusted for clarity in publication.

### Agarose gel electrophoresis and gel yield calculations

Gel characterization of DNA origami samples was performed using the Thermo Scientific™ Owl™ EasyCast™ B2 electrophoresis system. UltraPure agarose (Life Technologies, 16500500) was melted in 0.5× TBE (45 mM Tris, 45 mM boric acid, 0.78 mM EDTA, 11 mM MgCl_2_) to a concentration of 2.0% (w/v). The molten agarose was cooled and SYBR Safe was added (10 μl/160 ml of molten agarose). The samples were prepared in 10 μl volumes as follows: 5 μl of 6× AGLB [5 mM Tris, 1 mM EDTA, 30% (w/v) glycerol, 0.025% (w/v) xylene cyanol] loading dye, 250 fmol of the sample, and sterile water to reach the final volume. Control sample lanes were generally ∼0.5 μg of GeneRuler DNA Ladder 1 kb, 250–10 000 bp ladder (Thermo Fisher SM0311). The mixed samples were loaded onto the gel and separated for ∼2 h at 70 V at room temperature. Gel images were captured on a GE Typhoon FLA 9000 fluorescent imager using the SYBR Safe parameters as given in the Typhoon control software, with analysis of the images performed with FIJI ImageJ (v1.53c)(https://imagej.net/ij/index.html). Background subtraction with a rolling ball radius of 30–60 pixels was performed on linear TIFF images. The GelAnalyzer plugin in ImageJ and wand tool were used to integrate total pixel intensities from lanes of interest. DNA origami yields were determined by taking the ratio of the intensity of the band of interest with respect to all the species with a molecular weight larger than the excess staple strands.

### Agarose-gel-based extraction

Five parts of the folded DNA nanocube were combined with one part of 6× AGLB [5 mM Tris, 1 mM EDTA, 30% (w/v) glycerol, 0.025% (w/v) xylene cyanol] loading dye. The samples were loaded and run on agarose gels as described in the “Agarose gel electrophoresis and gel yield calculations” section. The bands were observed on a UV transilluminator and cut from the gel using a razor blade. The band slices were placed in a 15-ml tube and then thoroughly crushed using a large pestle (BioMasher V, Funakoski Co.). The tubes were inverted on a centrifuge and spun at 1.5 × *g* for 1 min, at which point the crushed gel could be transferred from the tube lid to a DNA spin column (Freeze ‘N Squeeze, Bio-Rad) with tweezers. The gel slice was further crushed using a small disposable pestle against the walls of the DNA spin column tube and then centrifuged at 7 000 × *g* for 5 min at room temperature. The flow-through solution containing the DNA structure was transferred to another collection tube. Next, the gel slices were again disturbed with the pestle, and the spin column again centrifuged as above, with the remaining flow combined into the other collection tube.

### CpG loading efficiency

The DNA origami was digested with DNase I so that the amount of nuclease-resistant CpG strand could be measured to determine the relative amount of the cargo loaded. Using Thermo Fisher DNase I, RNase-free kit (1 U/μl, EN0521), the reaction mixture was prepared in a 0.2-ml PCR tube by mixing the purified SQB (2 μg), 10× DNase I reaction buffer (1 μl), DNase I enzyme (1.5 μl), and water to attain a final 10 μl volume. Control samples with only the CpG staple oligonucleotides with and without DNase I were also prepared. The reactions were incubated at 37°C for 30 min to fully digest the sample. One part of the sample was mixed with one part of formamide loading buffer (2× solution of 95% formamide, 18 mM EDTA, 0.025% SDS, xylene cyanol, and bromophenol blue), which was then heated to 94°C for 2 min and cooled to 4°C until analysis on a gel. The samples were loaded onto a 15% denaturing polyacrylamide gel, which was prepared using the SequaGel UreaGel System (National Diagnostics, EC-833) and plastic 1.0-mm mini-gel cassettes (Invitrogen NovexTM, NC2010). Samples were migrated into the gel at 250 V for 45 min in 0.5× TBE (45 mM Tris, 45 mM boric acid, 0.78 mM EDTA) buffer. The gel was stained for 15 min in SYBR Gold (i.e. 3 μl of stain added to 15 ml of MilliQ water) on a horizontal shaker table in a dark room. Gel images were captured on a GE Typhoon FLA 9000 fluorescent imager using the SYBR Gold parameters as given in the Typhoon control software, with densitometry of the images performed with FIJI ImageJ (v1.53c) to determine relative CpG loading (Supplementary Fig. S5).

### Conjugation of nanocubes and barrels to the SQB

The purified SQB was mixed with either purified nanocube (at 10-fold stoichiometric excess to ∼50 nM SQB) or origami barrel (at 2-fold stoichiometric excess to ∼25 nM SQB) in 1× TEF buffer (5 mM Tris, pH 8.0, 1 mM EDTA, ∼12 or 10 mM MgCl_2_ for the nanocube and barrel, respectively). The samples were heated for 1 h at 37°C on a shaking incubator. In the case of samples conjugated to the nanocube, excess nanocubes were removed from the sample using PEG purification (as described for the SQB in the “PEG precipitation to purify origami from excess staples” section; Supplementary Fig. S7). Subsequently, images of the sample were captured by transmission electron microscopy (TEM) so that the number of bound cargoes on single particles could be counted manually.

### Slat folding and purification

Crisscross slats were originally introduced by Wintersinger *et al.* [19], whose assembly protocol we adapted for our own work. Unpurified dehydrated staple oligonucleotides were purchased from IDT at either 10- or 100-nmol scales and then rehydrated in water at a concentration of 200 or 500 μM. “Core staples,” i.e. those that are contained within the slat six helix bundle and do not bear cargo, were pooled together manually with an equal volume for each strand (a total of 127 staples). “Cargo” staples, i.e. those that occupy slots on the top (H5 helix) or bottom (H2 helix) of the slat, were pooled together using a Labcyte Echo 525 liquid handler (64 staples per slat). The different slats in this work all use the same set of core staples but differ in their cargo staple composition. For the base staple reuse test, most cargo staples were replaced with “control” staples that do not extend out from the slat core six-helix bundle. Only two cargo staples were introduced, one at each end of the slat. Both extend a single-stranded region with a unique sequence from the slat core, allowing them to be individually targeted with complementary binders. The Cadnano design file and all sequences used for the slat are provided in the Supplementary data, and the design is depicted graphically in Fig. 5.

After pooling, slat folding was conducted in 1× TEF buffer (5 mM Tris, pH 8.0, 1 mM EDTA) with 6 mM MgCl_2_, 50 nM p8064 scaffold (produced from M13 phage replication in *E. coli*), and ∼500 nM (10× excess over scaffold) of each staple strand. The following 18 h thermocycler protocol was used with a total reaction volume of 50 μl for each individual slat: raise temperature to 80°C for 10 min; ramp from 60°C to 45°C in 160 6.75 min steps reducing by 0.1°C per step; 16°C thereafter until collection of the sample for further processing and PEG purification.

Slats were either purified individually or in groups of 16 for the megastructure assembly. Before purification, the Mg^2+^ in the slat solution was increased from 6 to 20 mM by adding the appropriate volume of 1 M MgCl_2_. Subsequently, an equal volume of 2× PEG-purification buffer [5 mM Tris, 1 mM EDTA, 15% (w/v) PEG-8000, 510 mM NaCl] was added and mixed with the slat solution in a 1.5 or 2-ml DNA low-bind tube (Eppendorf). The mixture was then spun at 16 000 × *g* for 30 min, the supernatant was gently extracted using a pipette, and 40 μl (or 150 μl for the pools of 16 slats) of 1× TEF buffer with 20 mM MgCl_2_ added to the pellet (but not resuspended). The sample was spun once again (16 000 × *g* for 30 min), the supernatant was removed, and the final pellet was resuspended in 10–25 μl of 1× TEF + 10 mM MgCl_2_. The concentration of the final product was confirmed using NanoDrop after 1000 rpm shaking for 1 h at 33°C.

### Oridot folding and purification

The second origami design we tested was the “oridot,” a cuboid-shaped nanostructure originally intended to act as a high-density fluorophore carrier. The Cadnano design schematic and staple sequences have been provided in the Supplementary data. As with the other origami designs, all required staples were purchased unpurified and dehydrated. After rehydration, staples were grouped according to their functional moiety and length (exact details provided in the Supplementary data sequences file). The majority of these groups were PAGE-purified separately, apart from the “core” staples, which were used as is. To fold the origami, 20 nM p8064 scaffold was combined with each staple group in a 1× TEF + 12 mM MgCl_2_ buffer. The different staple groups were all added at five-fold excess apart from the “mini scaffolds,” which were added at a two-fold excess. Folding was carried out using the following protocol: Set to 80°C for 15 min, ramp from 60°C to 55°C at −0.1°C/2:42 min, then ramp from 55°C to 25°C at −0.1°C/2:45 min (total time: ∼16 h). PEG purification was handled in a similar fashion as the slats. However, we only did the two-step spin process for the first oridot folding run as we realized the second spin step was reducing the final origami yield. For all subsequent folds, we instead spun the PEG mixture only once and resuspended directly with 25 μl of 1× TEF + 10 mM MgCl_2_.

### Staple reuse of oridots and slats

After PEG purification, the remaining staples were collected for both the oridot and slat structures. Staple reuse was then conducted using the same protocol as defined for the SQB. Following staple recovery and concentration confirmation, new origami assemblies were folded with 1× staple replenishment as before. This procedure was repeated five times for both the base slat and oridot designs.

### Agarose gel electrophoresis and TEM analysis of slats and oridots

The same equipment and materials used to analyze the SQB were also used to assess the integrity of the slats and oridots. However, minor adjustments were made to accommodate the different designs. For gel electrophoresis, both origami designs were assessed in 100 ml of 1% 0.5× TBE + 11 mM MgCl_2_ agarose gels pre-stained with 1× SYBR Safe. A different volume for each origami was loaded to ensure the same concentration in each well (as calculated via NanoDrop measurement). The gels were run for 2 h at 80 V prior to imaging with the Typhoon scanner. For the fluorescent analysis, an unstained gel with the same formulation was used for analysis instead. For this unstained gel, the reference ladder and scaffold were premixed with 1× GelRed to allow for identification when using the Typhoon’s green laser. Two strands with slat cargo-binding sequences were purchased from IDT pre-attached to Cy5 and Cy3 fluorescent dyes on their 5′ end, respectively (sequences provided in the Supplementary data). Prior to gel loading, the two fluorescent strands were mixed with the slat origami at a concentration over 100× that of the slat origami. These were allowed to incubate together at 37°C for 30 min before mixing with loading dye and subsequent gel loading. For TEM staining and imaging, all origami samples were first shaken at 33°C/800 rpm for 30 min to loosen any aggregation, after which the slats and oridots were diluted to 0.4 and 1 nM in 1× TEF + 10 mM MgCl_2_, respectively. After glow discharging (15 mA/25 s), 4 μl of an origami sample was deposited onto FCF400-CU-50 grids and allowed to incubate for 2 min. After incubation, the sample was gently wicked off using filter paper and 4 μl of 0.5% uranyl formate was deposited. After a short incubation time of 2 s, the uranyl formate was forcefully blotted off using filter paper, resulting in positively stained particles. TEM imaging was conducted in the same manner as the SQB.

### Crisscross megastructure assembly

In a similar fashion to [19], we assembled a 450-nm square megastructure in a one pot reaction (design details provided in the Supplementary data). This involves adding a gridiron seed origami (gel purified and folded from a p8634 scaffold) to an excess of slat origami monomers designed to bind to each other in a specific pattern. Seed addition initiates assembly into the desired megastructure shape. All the slats were added in 8× excess of the seed concentration, which was set to 1 nM throughout all experiments. The buffer used for assembly contained 1× TEF, 15 mM MgCl_2_, and 0.01% Tween 20. The reaction was incubated in a thermocycler for 4 h at 45°C, after which the mixture was allowed to incubate at 37°C for 42 h. To test the staple reuse protocol, eight of the seed-binding (nucleating) slats were reused using the above-described methods. An additional square megastructure was then assembled, but this time using the new reused slats instead of the ones folded using the original staple solution.

### Crisscross bead pull-down

To remove excess slats and improve clarity of the final megastructures when imaging, we ran a bead-based purification protocol to selectively extract megastructures from the assembly solution. In brief, this involved capturing megastructures on beads through the hybridization of eight poly-A tails extending from select staples on the seed origami (sequence provided in the Supplementary data). Once contaminating slats are washed off, the megastructures can be detached from the beads through a toehold-mediated strand displacement reaction. The full protocol involved the following: (i) 100 nm streptavidin-coated beads (SV0100, Ocean Nanotech) were purchased, 200 μl were extracted, and their storage buffer exchanged to 100 μl of DNA-binding buffer (20 mM Tris, pH 7.5, 1 M NaCl, 1 mM EDTA, and 0.05% Triton X-100) using a magnet. (ii) 1.22 μl of 1 mM biotin–DNA in ultra-pure water (sequence provided in the Supplementary data) was added to the bead solution and allowed to incubate for 1 h at room temperature, rotating at 16 rpm to prevent the beads from settling. (iii) The mixture was washed three times with 200 μl of megastructure buffer (1× TEF, 15 mM MgCl_2_, and 0.01% Tween 20) before resuspension in 200 μl of the same buffer. (iv) The bead mixture was split in half, after which 19 μl of the megastructures formed with the original (Mega-O) and reused (Mega-R) slats were added to each fraction, respectively. (v) Both mixtures were allowed to incubate for 6 h at 37°C in a 16 rpm rotator. (vi) Both mixtures were washed three times with 400 μl of megastructure buffer, after which both were resuspended in 20 μl of displacement mix containing the megastructure buffer and 50 μM of invader DNA (sequence provided in the Supplementary data) to cleave off the megastructures from the beads. (vii) The final mixtures were allowed to incubate at 37°C (16 rpm) overnight before the magnetic beads were removed from each solution using a magnet. The final structures were shaken for 30 min at 33°C in a thermomixer (800 rpm) before preparation for TEM imaging using the positive staining approach described above.

## Results and discussion

We studied the folding of a DNA origami SQB used as a proof-of-concept vaccine nanoparticle in order to determine the viability of staple-strand reuse [6]. The dimensions of the SQB are ∼35 nm × ∼27 nm × ∼22.5 nm, with its square-lattice arrangement presenting 126 modifiable 3′ and 5′ helical ends, which can be appended with therapeutic cargoes. The flat face in Fig. 1B(i) displays 18 CpG immunostimulant strands with 3.5 nm spacing, recognized as the optimal spacing for toll-like receptor 9 (TLR9) interaction [6, 20–23]. We note that we employ CpG segments bearing a non-natural phosphorothioated backbone so as to protect these sequences from nuclease digestion for *in vivo* environments. Conversely, the jagged face in Fig. 1B(ii) has ssDNA handle strands to bind Cy5 fluorophores and other cargoes for downstream experiments.

**Figure 2.**
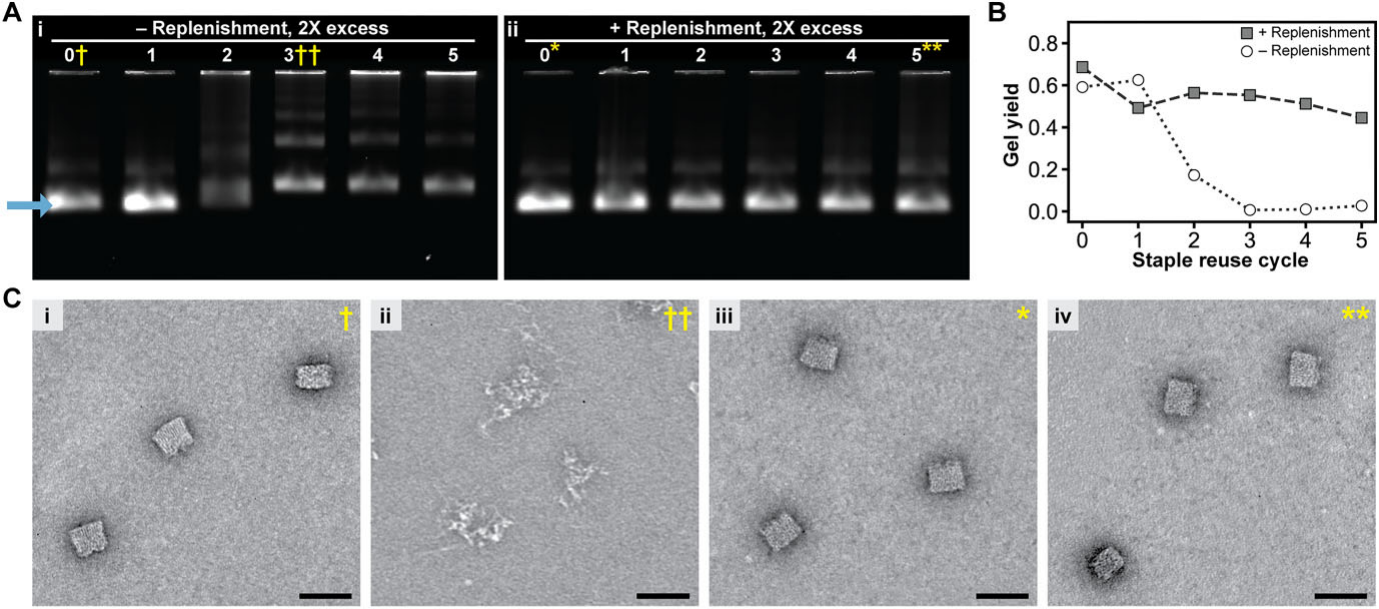
Replenishment of staple strands in excess with fresh strands enables successful folding of the SQB DNA origami with multiple cycles of reuse. (**A**) Agarose gel analysis of the effect of staple reuse on SQB DNA origami folded with a 2× excess of staple strands. In gel (i), the staple reuse was conducted without replenishment. A significant decrease in the mobility of the structure was observed after the second cycle of reuse, with an eventual complete loss of the output origami band. In contrast, gel (ii) shows the result after conducting staple reuse with replenishment of the folded staples after each round. The results showed minimal differences in the origami’s gel mobility between each folding cycle, confirming that staple strand replenishment maintains structural consistency. (**B**) Yields of the desired monomer structure, with the yield determined from densitometry of the SQB band (i.e. arrow bottom left corner of Fig. 2Ai) with respect to the overall well. (**C**) Representative negative-stain TEM micrographs of the samples indicated in panel (A), which confirmed the results we observed with gel electrophoresis. All scale bars shown represent a length of 50 nm; large fields of TEM images can be found in the related Supplementary section.

We first tested folding of the SQB through five cycles of reuse of the staple strands, in this case using a two-fold molar excess of the latter over scaffold strand in each cycle sans any replenishment with fresh staple oligonucleotides. The volume of each folding reaction with reused staple strands was lessened to maintain the relative two-fold excess of staple strands. As shown on the agarose gel in Fig. 2A(i), the SQB has similar mobility after a single cycle of reuse as compared to the origami when folded only with fresh staple strands. However, there is a drastic slowdown in the gel mobility of the structure by the second cycle of reuse, which becomes even more extreme by the third, fourth, and fifth rounds. Gel densitometry indicated that the relative yield of the SQB monomer was diminished from ∼60% to ∼20% between the first and second reuse cycles, and finally with negligible yield after additional reuse cycles (white circular data points on Fig. 2B, see the “Agarose gel electrophoresis” section for details about yield calculations). Using negative stain TEM, we concluded that well-formed SQB particles could not be observed by the third cycle of staple reuse without replenishment (Fig. 2C, i and ii). These observations suggest that the origami folding process preferentially selects full-length strands from a folding solution containing truncated staple oligonucleotides; a similar argument has been made earlier to rationalize the observation that a large excess of synthetic staple strands is needed for high-quality folding of DNA origami more generally [2]. We concluded that the staple pool was gradually enriched with undesirable flawed strands with continued cycles of reuse, to the extent that it could no longer allow satisfactory folding of the SQB.

To see whether staple strands with consecutive cycles of reuse could be used to properly fold the SQB, we repeated the aforementioned experiment with replenishment of these strands. After each folding using a two-fold excess of staple strands, the excess strands were collected and combined with one equivalent of fresh strands to replace those that were incorporated into the origami in the previous folding round. On an agarose gel, we observed similar mobility of the SQB regardless of whether it was folded with fresh staple strands versus whether the strands were reused and replenished up to five times, with ∼60% yield of the desired structure (Fig. 2A, ii, and gray square data points in Fig. 2B). While there did seem to be a gradual and slight diminishing of the yield with more reuse of the staple strands (e.g. ∼45% by the fifth cycle), observation of single SQB particles by TEM indicated qualitatively similar folding between the initial and last cycles of reuse (Fig. 2C, iii and iv). Taken together, these observations suggest that replenishment of the recovered staple-strand pool with fresh strands can sufficiently restore these pools over multiple cycles of reuse so that they can satisfactorily enable the folding of the SQB.

**Figure 3.**
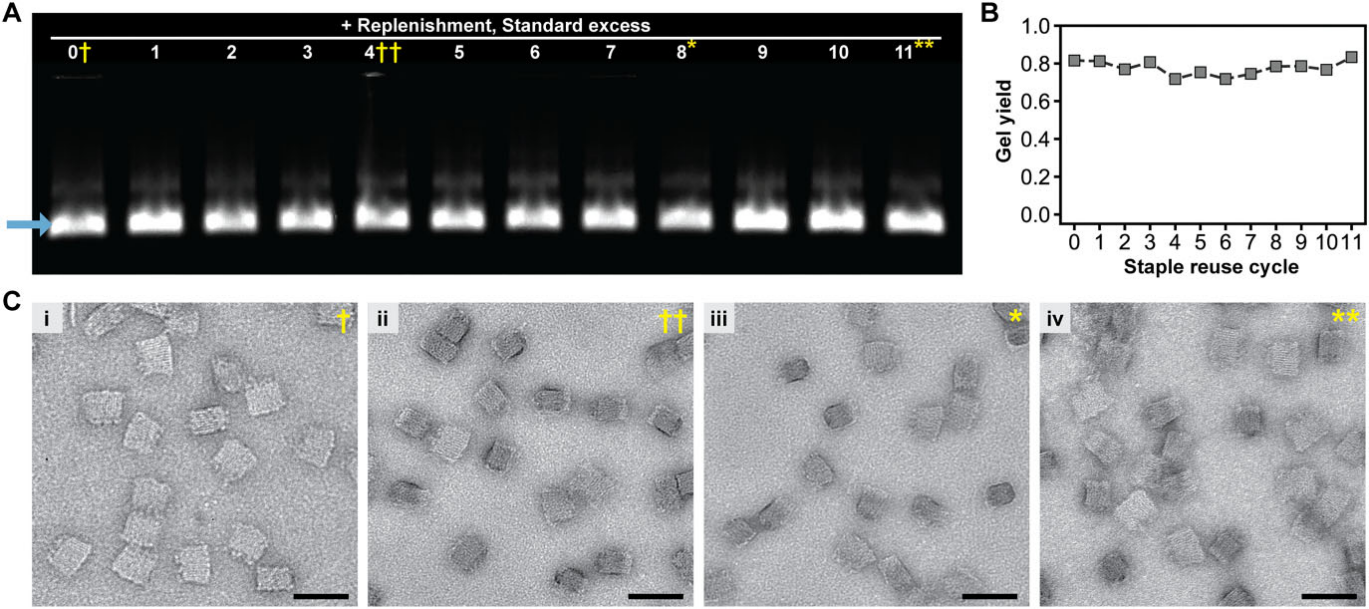
(**A**, **B**) Agarose gel showing that folding yield is sustained with replenishment of reused staple strands when there is at least a 5× excess of staple strands, with the yield determined from densitometry of the SQB band (i.e. arrow left bottom corner of Fig. 3A) with respect to the overall well. (**C**) Representative negative-stain TEM micrographs showing single folded SQB particles of the samples noted on the gel in panel (A). Scale bars are 50 nm; large fields of TEM images can be found in the related Supplementary section.

Having shown the viability of folding origami with staple strands that were in excess, we wanted to see whether satisfactory folding could be attained over a greater number of folding cycles. We hypothesized that larger excesses of staple strands would be more robust to depletion of full-length strands over continued reuse because there would be a larger total starting pool of oligonucleotides. We note that we initially focused on a mere two-fold excess of staple strands over scaffold for easier testing of our hypothesis about the eventual need for replenishment (i.e. to make this more evident with fewer folding cycles required). Given that larger excesses of staple strands increase the likelihood that 3′ and 5′ features are exhibited [12] and the importance of high incorporation rates of such features for making the effect of an origami therapeutic reliable and repeatable, we folded the SQB using the following molar excesses as determined by their therapeutic importance: (i) immunostimulant CpG staple strands at 20-fold excess; (ii) ssDNA handle strands for Cy5 fluorophores and other potential cargos (eg. antigens) at 10-fold excess; and (iii) core strands for the structural integrity of the SQB at 5-fold excess (Supplementary Table S1). Indeed, reuse and replenishment of staple strands could sustain satisfactory folding of the SQB with ∼80% yield across 11 cycles of reuse, as shown by the agarose gel and densitometry of the gel in Fig. 3A and B. Single SQB particles were qualitatively similar when folded using fresh staples, versus with staples reused 4, 8, or 11 cycles, as shown in the TEM micrographs in Fig. 3C (i– iv). We also note that yield of SQB was noticeably diminished after more than six cycles of reuse with no replenishment using the standard staple excesses noted above, when the total volume of reaction was not adjusted to maintain the standard excess of staple strands with respect to the scaffold (Supplementary Fig. S4).

**Figure 4.**
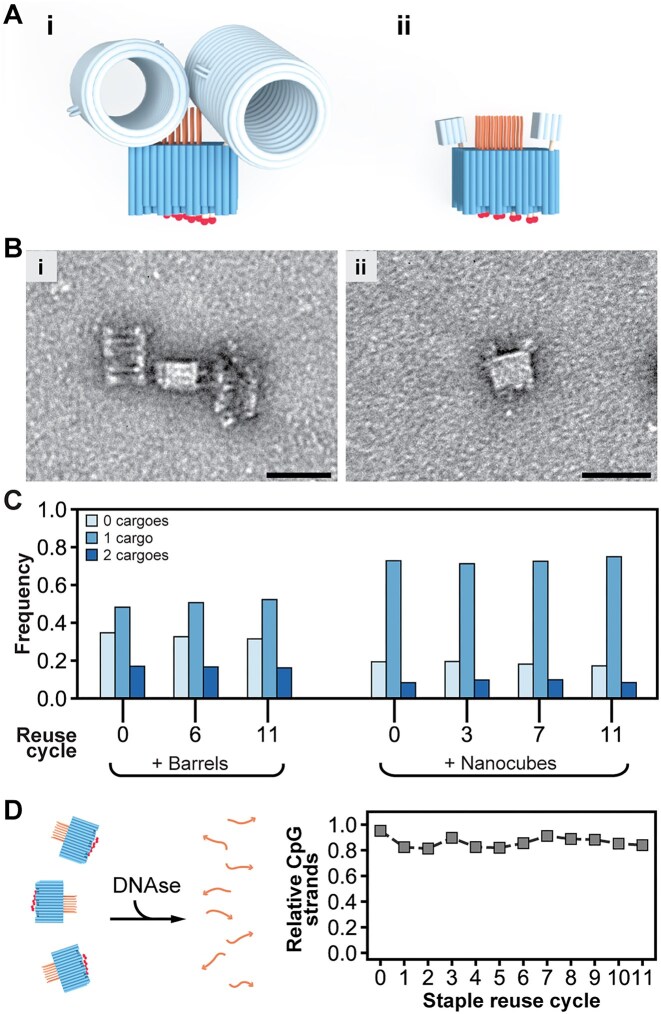
Programmed features on the staple-strand ends are successfully displayed on DNA origami therapeutics folded from reused strands with replenishment. (**A**) (i, ii) Models of the barrel and nanocube cargoes attached to the SQB to assess incorporation efficiency of two handle strands. (**B**) Representative TEM micrographs of (i) barrels and (ii) nanocubes attached to the SQB. (**C**) The relative frequency with which zero, one, or two cargoes were observed in TEM micrographs of single SQB particles made from reused staple strands. *N*_particles_ = 570, 612, and 549 for SQB0, SQB6, and SQB11, respectively for the barrels. *N*_particles_ = 1100, 1288, 1821, and 1521 for SQB0, SQB3, SQ7, and SQ11, respectively for the nanocubes. (**D**) The relative fraction of CpG strands remaining after DNase digestion of SQB samples made with reused staple strands. Large fields of TEM images can be found in the related Supplementary section.

To determine whether there was any difference in the staple incorporation and folding quality with reused staple strands, we measured the presence of features on the origami. We initially examined a pair of 3′ ssDNA 16-nt handles on the extreme corners of the flat face of the SQB (i.e. the short light orange squiggles in Fig. 1B, i). We created two distinct cargoes with a complementary 3′ ssDNA antihandle, including a DNA origami barrel [15] and a DNA nanocube [16]. As an example, we selected various purified SQB samples (depicted in Fig. 2D) for incubation: specifically, for the nanocubes, we chose SQBs 0, 3, 7, and 11, whereas for the barrels, we opted for SQBs 0, 6, and 11. We indirectly quantified the incorporation of the handle on the SQB by counting the number of cargoes conjugated to single particles in TEM images (Fig. 4A and B). Encouragingly, the relative conjugation of either the nanocube or barrel to the SQB was similar regardless of the number of times that the staple strands were reused and replenished (Fig. 4C). For instance, about ∼73% and ∼9% of the particles were bound with one and two nanocubes, respectively (Supplementary Tables S7 and S8), with this remaining nearly constant for the DNA origami folded using fresh staple strands versus those reusing the strands in 3, 7, and 11 consecutive cycles.

We used the pairwise conjugation data in Fig. 4C to compute the probability that a cargo was attached to a single handle (see Supplementary Text S2). We determined there was ∼40% chance that a barrel would be bound to one of the handles tested on the SQB, irrespective of whether the origami was folded from fresh or reused staple strands. This computed probability might be representative of the incorporation efficiency of staple strands and their 3′ features on the origami, though there are several confounding factors that make it difficult to judge the true incorporation efficiency of all the single staple strands individually from solely this data, as explained in Supplementary Text S2. Nonetheless, the similarity in the relative conjugation frequencies for the SQB made using reused strands to its counterpart made with fresh staple strands suggests similar incorporation of the 3′ handle across all the samples tested. These observations suggest that origami folded using staple strands that were reused multiple times could be of sufficient quality for the validation of therapeutic DNA origami designs.

To determine whether there was any difference in the staple incorporation between the samples in the various reuse rounds, we conducted an assay to measure the presence of CpG, one of the most important features of the SQB vaccine. The CpG strands we use for the SQB were synthesized with a phosphorothioate backbone, which allows them to resist enzymatic degradation from DNases. This feature gives us the opportunity to perform a quantitative assay in the presence of this oligodeoxynucleotide on the SQB. We incubated the samples with DNase (see the “CpG loading efficiency” section) and this enzyme digested all the origami leaving in solution just the strands of CpG (see Supplementary Fig. S5). This was then quantified through gel densitometry giving as a result a stable rate of incorporation differing around 15% between the cycles (Fig. 4D).

To gain intuition of why reused strands could make origami, we created a stochastic model to determine how staple excesses, impurities, and replenishment might influence the overall quality of the strand pool (Supplementary Text S3). We tested these experimental parameters in the model and qualitatively compared the results to the data in Fig. 2. From inspection of Fig. 2, we concluded that there is some amount of bias favoring the incorporation of full-length staple strands during origami folding. Quantitative measurements of exactly how much the staple pool was degrading was beyond the scope of this manuscript. However, our stochastic model provides intuition of how strand quality might degrade through such cycling (Supplementary Fig. S6A and B). Our stochastic model also illustrates how replenishment is critical to preserve the purity of the staple strands to yield origami with large proportion of full-length staple strands over multiple cycles of reuse, and how larger excesses of staple strands when they are not replenished after each folding could slow the lessening of the quality of the origami over sustained reuse (Supplementary Fig. S6C and D, leftward plots). Conversely, our model illustrates how replenishment is able to largely preserve the quality of the staple pool over multiple cycles of reuse regardless of whether the modeled folding is conducted with 2-, 5-, or 10-fold excesses of staple strands (Supplementary Fig. S6C and D, rightward plots).

**Figure 5.**
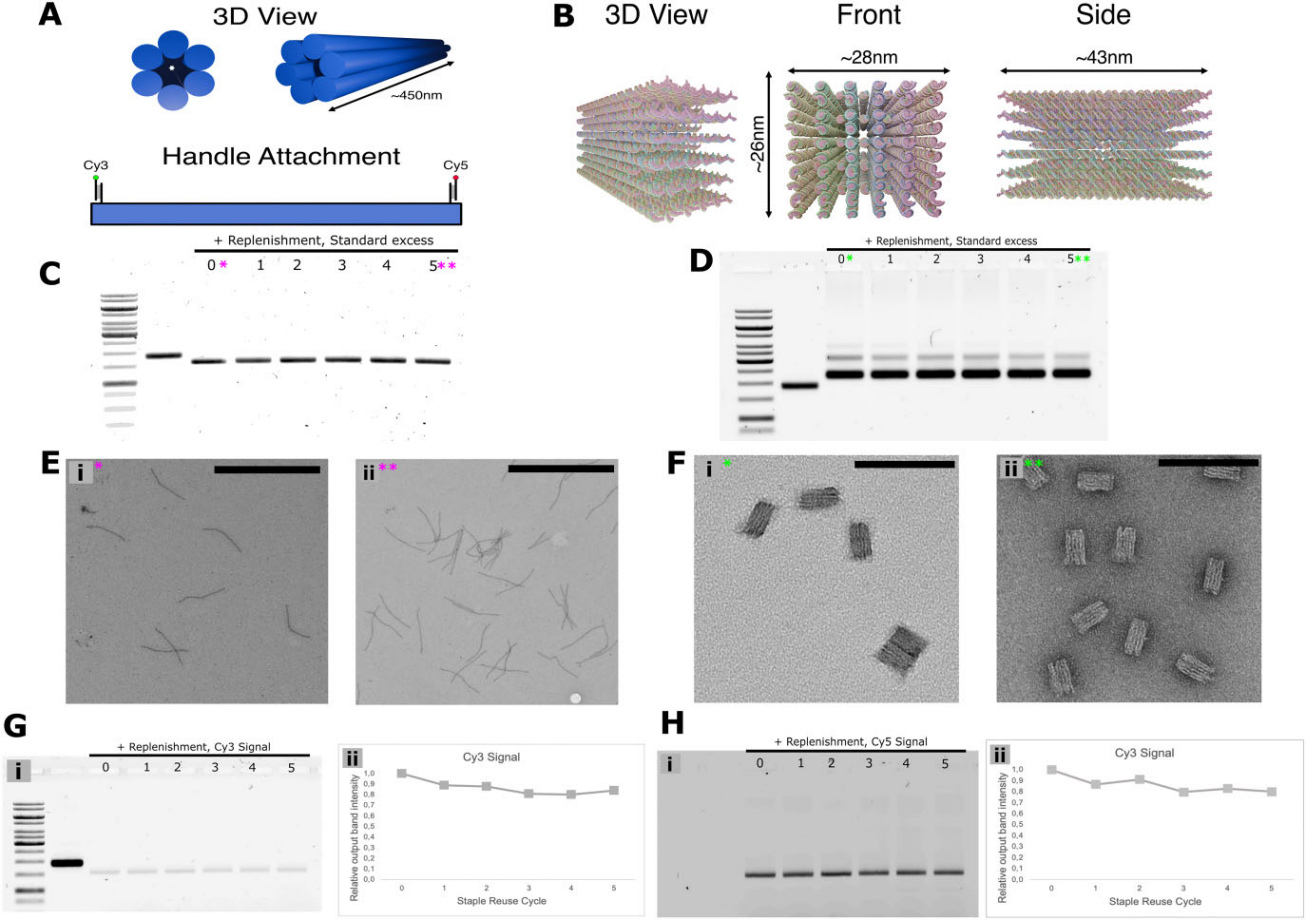
Validation of the proposed method with additional DNA origami designs. (**A**) 3D representation of the six-helix bundle slats used for crisscross origami, along with a schematic showing the positioning of cargo strands for fluorophore attachment. (**B**) 3D representation of the oridot origami structure. (**C**) Agarose gel analysis (SYBR Safe stain) of purified crisscross slats incrementally folded with the same reused staple mixture (with replacement) using our methodology. The numbers represent the number of times the staple mixture was reused before being used to fold the corresponding sample. (**D**) Agarose gel analysis (SYBR Safe stain) of purified oridots incrementally folded with the same reused staple mixture (with replacement) using our methodology. (**E**) TEM images illustrating the integrity of the slat samples after (i) the initial folding and (ii) the fifth reuse round (scale bar represents a length of 1 μm). (**F**) TEM images illustrating the integrity of the oridots after (i) the initial folding and (ii) the fifth reuse round (scale bar represents a length of 100 nm). (**G**) Agarose gel analysis of purified crisscross slats after five reuse rounds showing (i) the fluorescence recorded from Cy3 fluorophores conjugated to one end of the slat and (ii) the quantification of the bands representing the intensity of the conjugated fluorophores. (**H**) Agarose gel analysis of purified crisscross slats after five reuse rounds showing (i) the fluorescence recorded from Cy5 fluorophores conjugated to the opposite end of the slat and (ii) the quantification of the bands representing the intensity of the conjugated fluorophores. Both panels (G) and (H) are images of the same unstained gel. The ladder and scaffold in the first and second lanes show up in the Cy3 channel as they have been sample-stained with GelRed to act as reference markers. For both fluorophores, the output signal remained consistent throughout each folding cycle (within the typical gel electrophoresis error range). Large fields of TEM images can be found in the related Supplementary section.

To further validate the staple reuse paradigm, we prepared, folded, and reused two additional DNA origami structures employing a different scaffold and markedly distinct shapes (Fig. 5A and B). The first design selected was the “oridot,” a compact structure consisting of single-layer scaffold blocks held together by 100-mer “mini-scaffold” staples. The second is the standard six-helix bundle or “slat,” which has been used as the basic building block for creating micron-sized megastructures using the crisscross origami technique [20]. For both new origami designs, we first folded the structure using the staple excesses defined in the “Materials and methods” section, and then proceeded to refold the structure after retaining the excess staple mixture following PEG purification. This process was repeated five times, every time replenishing just the depleted staples following each successful fold. The aforementioned 100-mer mini-scaffolds were also reused along with the rest of the staples for the oridot design.

As with the SQB, we performed both agarose gel electrophoresis and TEM imaging to assess the folding results after each round of staple reuse. Both new origami designs continued to retain their characteristic electrophoretic patterns after each reuse round, and single particles imaged via TEM confirmed the lack of variation between each round (Fig. 5C–F). Furthermore, we included a unique handle strand on each end of the slat origami design. After completing all five folding rounds, we assessed the incorporation of the unique handles at each round by measuring the uptake of fluorophores conjugated to strands complementary to each handle. As before, fluorophore uptake was consistent across the board for both ends of the origami, providing further evidence of the integrity of origami structures folded using reused staples (Fig. 5G and H).

Finally, we stress-tested our approach by folding megastructures; hierarchical DNA origami structures formed out of many slat monomers, using reused slats [19]. In crisscross origami, 32 staples on each slat have unique 7-mer handles on their 3′ ends, so that a given slat can engage with other slats to form a higher-order structure composed from many origamis. Without these 7-mer handles, crisscross origami megastructures will not assemble, and thus act as an ideal testing system for our reuse method. To conduct our test, we designed a square-shaped 450 nm × 450 nm megastructure composed of 64 slats, from which we selected eight of the nucleating slats as our reuse targets (Supplementary Fig. S8A). The selected slats contained the aforementioned 32 handle sequences, along with 5 additional 10-mer seed binding handles used to initiate assembly. After refolding these slats with a single staple reuse round, we assembled megastructures using the original slats and prepared two samples: one containing the eight original slats and another with the eight reused slats. After assembly and purification, the TEM images of Supplementary Fig. S8B and C show that the reused slats have correctly assembled into megastructures of the same quality and yield as those formed from the original slats. Taken together, these extended test results continue to confirm the robustness and applicability of our method, regardless of the origami design, scaffold, or staple length.

## Conclusion

This study showed that a DNA origami structure could be properly folded when reusing the excess staple oligonucleotides from prior folding experiments, so long as the staple strands were replenished with an equivalent amount of fresh staple strands to compensate for those lost during the previous folding experiment and the DNA ethanol purification process. We synthesized a proof-of-concept DNA origami therapeutic with satisfactory incorporation of cargoes by reusing the excess staple strands up to 11 times. A simplistic model of folding illustrates the extent to which the experimental process could bias the selection of full-length strands into the origami, which could explain why we observed high-quality product formation despite the presumed gradual decrease in the purity of the staple pool. Reuse and replenishment of staple strands can significantly lessen the amount of staple strands that must be purchased to fold DNA origami. In turn, this could significantly lessen the costs of folding milligram amounts of DNA origami that would be needed to prototype and validate potential origami therapies in laboratories.

## Supplementary Material

gkaf527_Supplemental_Files

## Data Availability

The data underlying this article are available in the article and in its online supplementary material. The code for the plotted graph is available from https://doi.org/10.5281/zenodo.15227436. Folding sequences are available from previous citations.
